# Epidural Analgesia With Bupivacaine and Fentanyl Versus Ropivacaine and Fentanyl for Pain Relief in Labor: A Meta-Analysis

**DOI:** 10.1097/MD.0000000000000880

**Published:** 2015-06-12

**Authors:** Shanbin Guo, Bo Li, Chengjie Gao, Yue Tian

**Affiliations:** From the Department of Pharmacy, Shengjing Hospital of China Medical University, Shenyang (SG); Department of Anesthesiology, Jinan General Hospital, PLA Jinan Military Area Command, Jinan (BL, CG); and Department of Anesthesiology, Shengjing Hospital of China Medical University, Shenyang, China (YT).

## Abstract

The aim of this study was to compare the efficacy and safety of the combinational use of bupivacaine and fentanyl versus ropivacaine and fentanyl in epidural analgesia for labor.

Multiple electronic databases were searched by using appropriate MeSH terms, and keywords for original research papers published before October 2014. Meta-analyses were based on mean differences between the groups as well as odds ratios. Statistical heterogeneity was tested by I^2^ index.

Fifteen randomized controlled trials, recruiting 2097 parturient mothers overall, were selected for the meta-analyses. Concentrations of the preparations used (weight/volume; mean and standard deviations) were bupivacaine 0.1023% ± 0.0375%, ropivacaine 0.1095% ± 0.042%, and fentanyl 0.00021% ± 0.000089%. There were no statistically significant differences between both the combinations in the mean change in Visual Analog Score for pain during labor, incidence of instrumental or cesarean delivery, neonate Apgar score of <7, maternal satisfaction, duration of either first or second stage of labor, oxytocin use for induction, onset of analgesia, and duration of analgesia. Women who received ropivacaine and fentanyl had significantly lower incidence of motor blocks (odds ratio [95% CI] = 0.38 [0.30, 0.48] *P* < 0.00001, fixed effect and 0.38 [0.27, 0.54] *P* < 0.0001, random effects I^2^ 30%) when compared with women who received bupivacaine and fentanyl. Incidence of side effects was similar for both the combinations.

Analgesia with ropivacaine in combination with fentanyl at 0.1%:0.0002% ratio for labor pain relief is associated with lower incidence of motor blocks in comparison with analgesia with bupivacaine and fentanyl at similar ratio (0.1%: 0.0002%).

## INTRODUCTION

Analgesic adequacy during labor along with the avoidance of adverse effects is vital for obstetric conditions. Painful labor can have negative impacts on maternal and fetal physiology. In neuraxial analgesia, the analgesics are injected or infused in close proximity to the spinal cord by using catheter, usually either intrathecally into the cerebrospinal fluid or epidurally into the fatty tissues around the dura, to block nerves that transmits pain signals to the brain.^1,2^ Much lower pain scores with least adverse effects on maternal cardiovascular or pulmonary functions and fetal physiology with higher maternal satisfaction are reported with the use of neuraxial analgesic techniques during labor and delivery.^3^

Epidural administration of amide local anesthetics in combination with opioids is widely used for pain relief in labor because of the dose minimizing and side effects reducing benefits.^4–6^ Bupivacaine is the most widely used long-acting amide local anesthetic. It is a racemic mixture of 2 stereoisomers. Ropivacaine, a levorotatory propyl homologue of bupivacaine, because of its structural features and physicochemical properties, is found to be less toxic to nervous system and heart in comparison with bupivacaine, although, it possesses relatively lower potency.^4,7^ Fentanyl, a low molecular weight, high potency, and lipid soluble synthetic opioid, is a suitable analgesic drug which is in use for labor since many decades.^8^

Previously, the efficacies of epidural analgesia for labor with bupivacaine and ropivacaine have been reviewed, and the outcomes were found similar for both the drugs except for a statistically untested (because of higher heterogeneity) evidence of higher incidence of motor blocks in bupivacaine-treated women.^9^ Recently, the efficacy and safety of bupivacaine in combination with sufentanil have been reviewed against levobupivacaine and ropivacaine both in combination with sufentanil where it has been observed that the incidence of motor blocks was nonsignificantly higher in the bupivacaine–sufentanil combination.^10^ So far, there is no systematic study to review the clinical trials that examined the efficacy and safety of these local amides in combination with fentanyl. Purpose of this meta-analysis is to compare the efficacy and safety of the combinational use of bupivacaine and fentanyl with ropivacaine and fentanyl in epidural analgesia for labor pain relief by analyzing data generated in the relevant randomized controlled trials (RCTs).

## METHOD

This meta-analysis is carried out by following Preferred Reporting Items for Systematic Reviews and Meta-Analyses guidelines. Table 1 summarizes important features of the method used to carry out the present study. Meta-analysis does not involve ethical review.

**TABLE 1:**
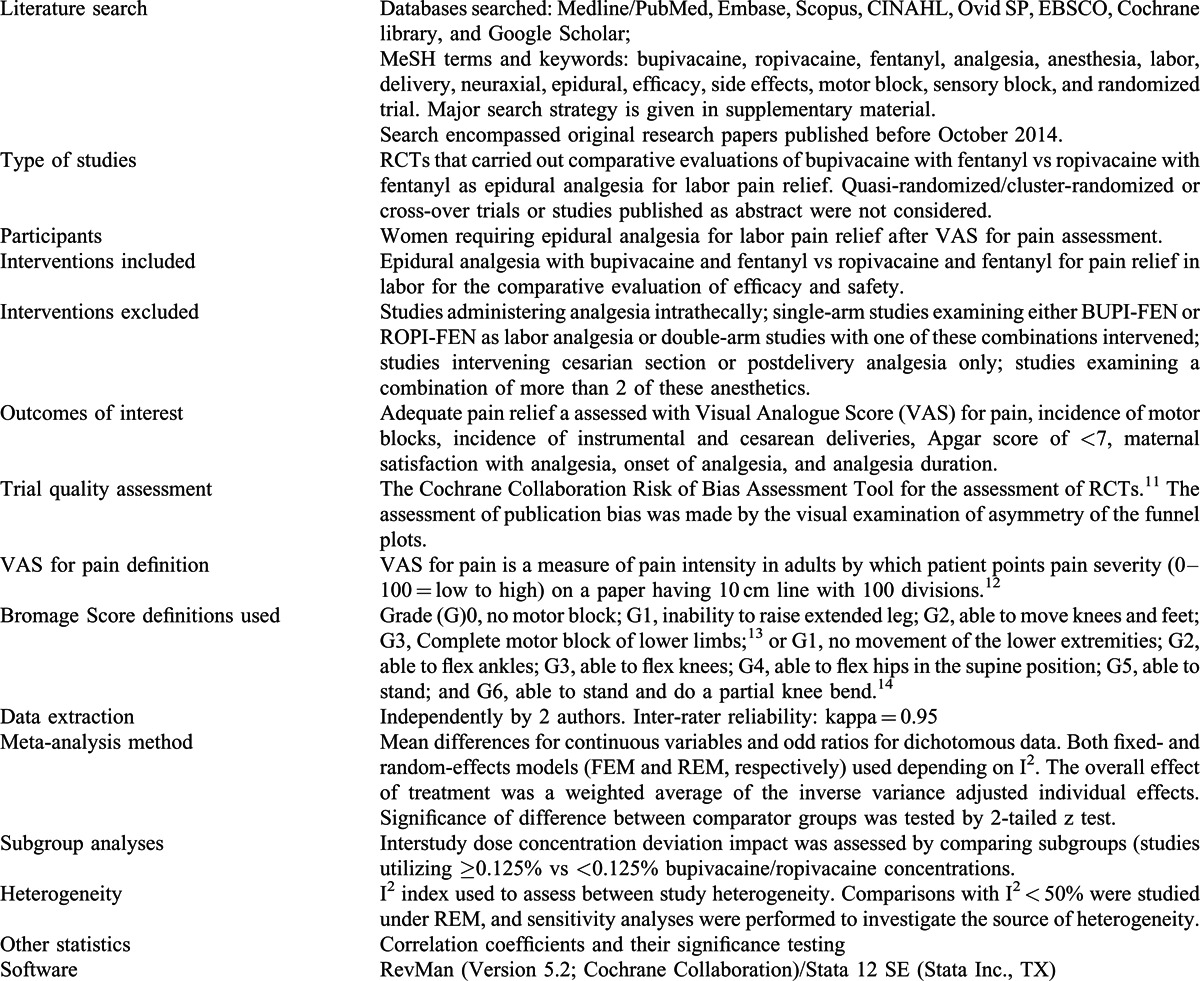
Important Features of the Method Used for the Present Study

### Inclusion and Exclusion Criteria

The inclusion criteria were, RCTs, recruiting women in labor to study the efficacy and safety of epidural analgesia with bupivacaine versus ropivacaine, both in combination with fentanyl (hereinafter BUPI-FEN and ROPI-FEN, respectively); have compared at least 3 efficacy and/or safety parameters; the combined analgesic solution infused epidurally to maintain analgesia during labor and delivery; and the effectiveness of analgesia has been assessed with Visual Analog Score (VAS) for pain, and the participants entered the trial after being assessed with VAS for pain and were found appropriate for the trial. The exclusion criteria were: studies administering analgesia intrathecally; single-arm studies examining either BUPI-FEN or ROPI-FEN as labor analgesia or double-arm studies with one of these combinations intervened; studies evaluating BUPI-FEN and/or ROPI-FEN combinations as cesarean section or postdelivery analgesia only; and studies examining a combination of more than 2 of these anesthetics.

### Data Extraction, Synthesis, and Statistical Analysis

The data were obtained from the published research papers of respective trials and were organized in datasheets. Data regarding the participants’ demographic and obstetric characteristics, interventions, and outcomes were extracted independently by 2 reviewers. For meta-analyses, mean and standard deviations (MSD) of the comparator variables of interest were used to calculate the mean differences along with 95% confidence intervals (95% CI) for each constituent study, which then led to the calculation of overall effect size. For dichotomous variables, meta-analyses were based on odds ratios. Statistical heterogeneity was tested by I^2^ index. Correlation coefficients between the dose concentrations and important endpoints with their significance levels were calculated by using Stata 12 software. Visual examination of the funnel plot was used as proxy measure to judge selection biases, and sensitivity analyses were performed to investigate the source of higher heterogeneity in comparisons with I^2^ > 50%.

## RESULTS

**FIGURE 1:**
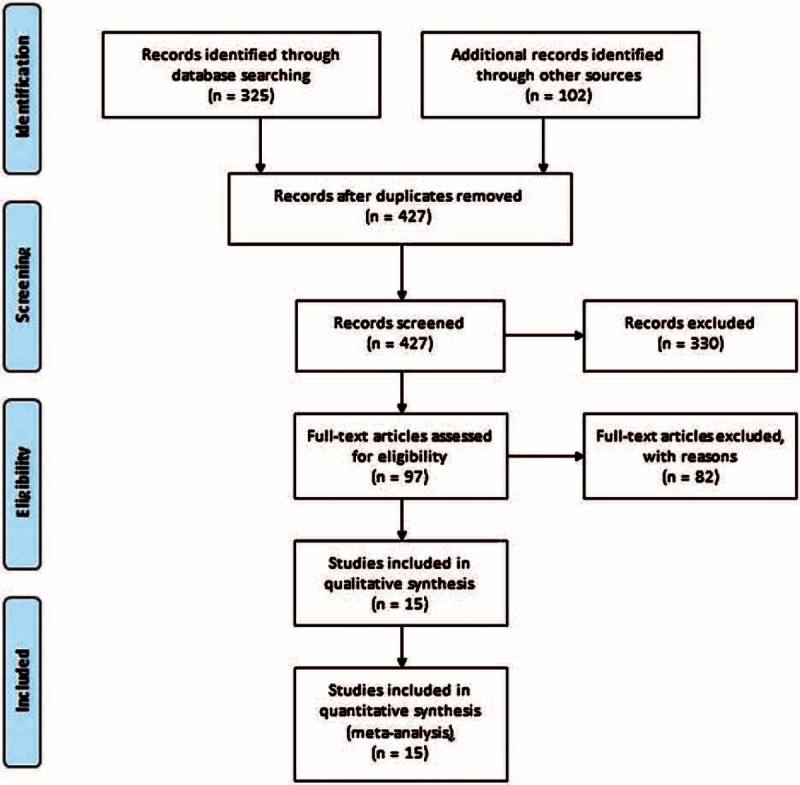
Flowchart of literature screening and study selection process.

Fifteen studies^15–29^ were selected for the meta-analyses by following the inclusion and exclusion criteria. A flowchart of literature retrieval, screening, and study selection process is presented in Figure 1, and the characteristics of the included studies are presented in Table 2  . Overall, the included studies recruited 2097 women in labor. Age (MSD) of the participants ranged between 22.9 ± 0.6 and 31 ± 4 years. Height and weight of the parturient women as MSD ranged between 159.7 ± 2.73 to 167 ± 7 cm and 64.2 ± 4 to 84 ± 13 kg, respectively.

**TABLE 2:**
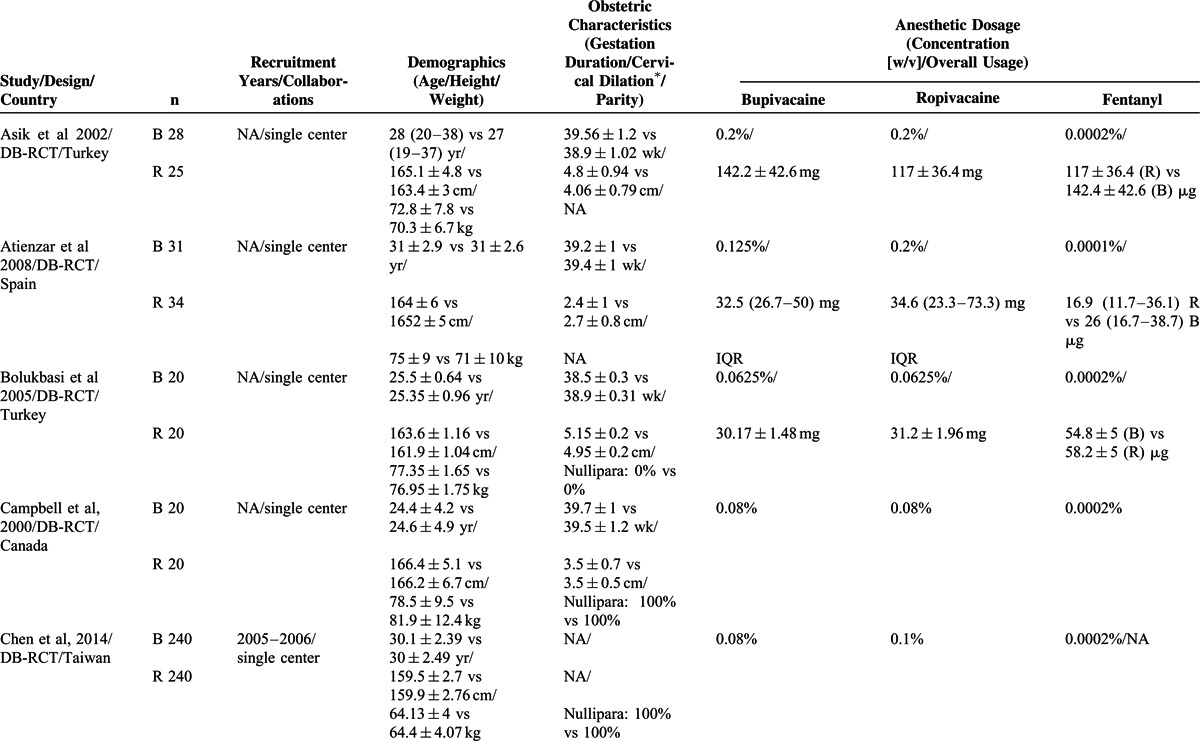
Important Characteristics of the Included Trials

**TABLE 2 (Continued):**
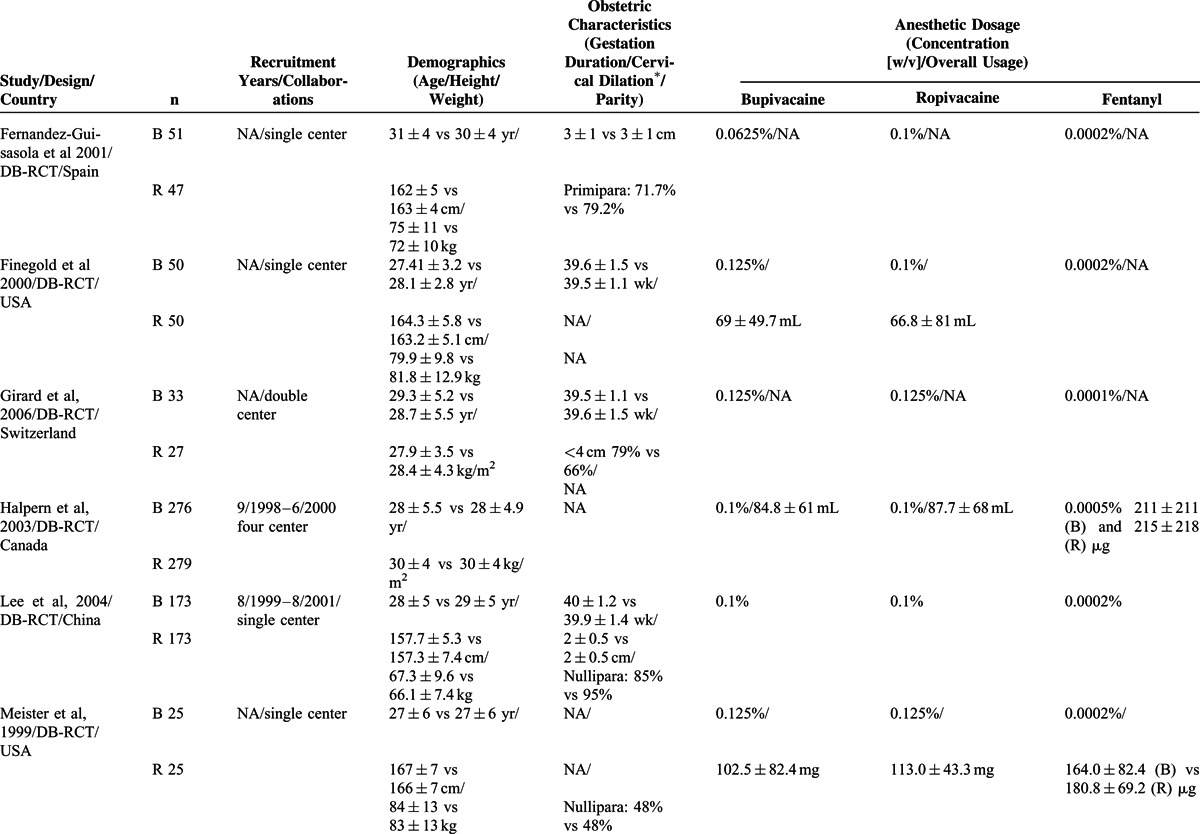
Important Characteristics of the Included Trials

**TABLE 2 (Continued):**
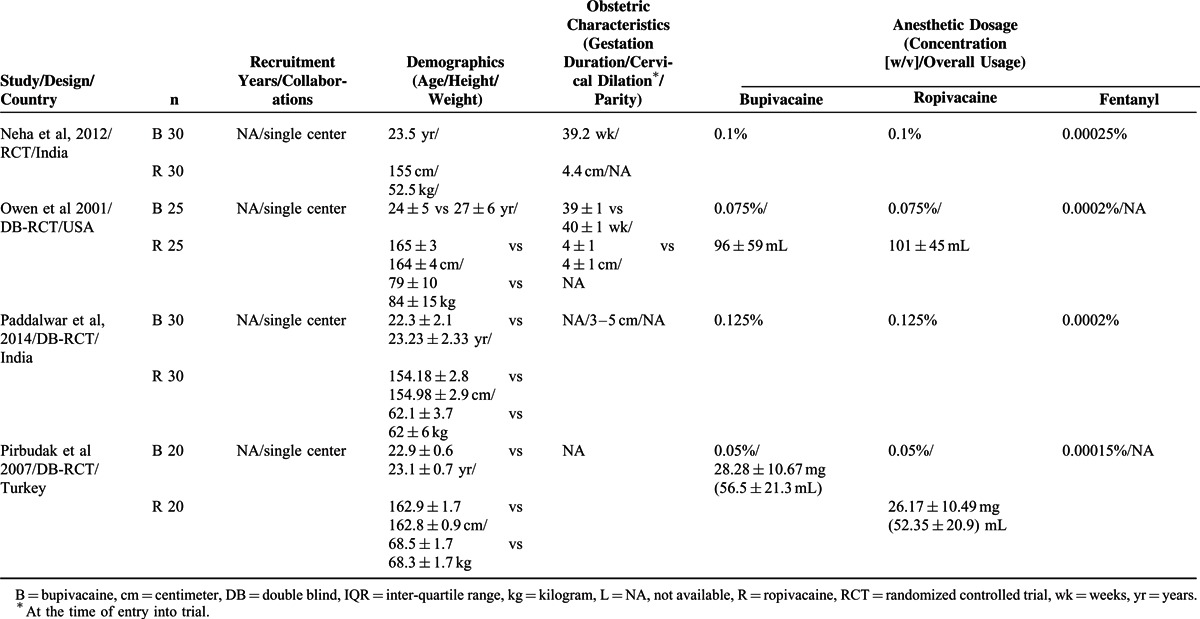
Important Characteristics of the Included Trials

Among the important obstetric data as MSD, gestation period ranged between 38.6 ± 0.3 and 39.6 ± 1.1 weeks and cervical diameter at the time of entry into the trial ranged between 2.4 ± 1 and 5.15 ± 0.2 cm. Concentrations of the preparations used (weight/volume, MSDs) in this population were: bupivacaine 0.1023% ± 0.0375%, ropivacaine 0.1095% ± 0.042%, and fentanyl 0.00021% ± 0.000089%. Loading dose volume was 10.4 ± 4.2 (5–20) mL, loading time 15.7 ± 8.6 (5–30) minutes, and maintenance dose volume of the study drugs was 8.8 ± 3.6 (4–15) mL/hour. Studies utilizing patient controlled epidural analgesia (PCEA) systems had locktime of 12.5 ± 4.3 (10–20) minutes and 8 ± 2 (6–10) mL/hour infusion rate. All studies utilized study drugs for loading except for one that utilized 0.7% lidocaine with fentanyl as loading dose followed by study drugs for the maintenance of analgesia.^20^

**FIGURE 2:**
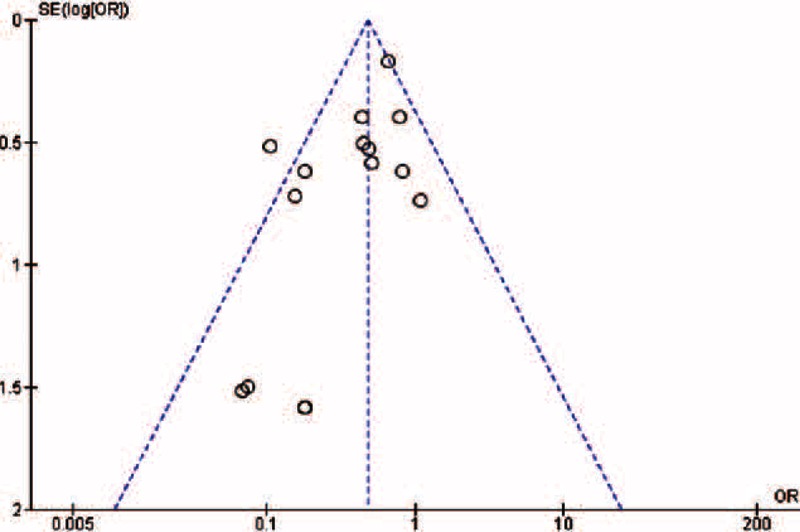
Funnel plot, corresponding to the meta-analysis of motor block incidence data, showing a low-level publication bias.

The quality of the included trials was generally good when assessed against the trial manifesto of each study (Table 3). A low-level selection bias including publication bias was also evident from the visual examination of the funnel plot (Figure 2).

**TABLE 3:**
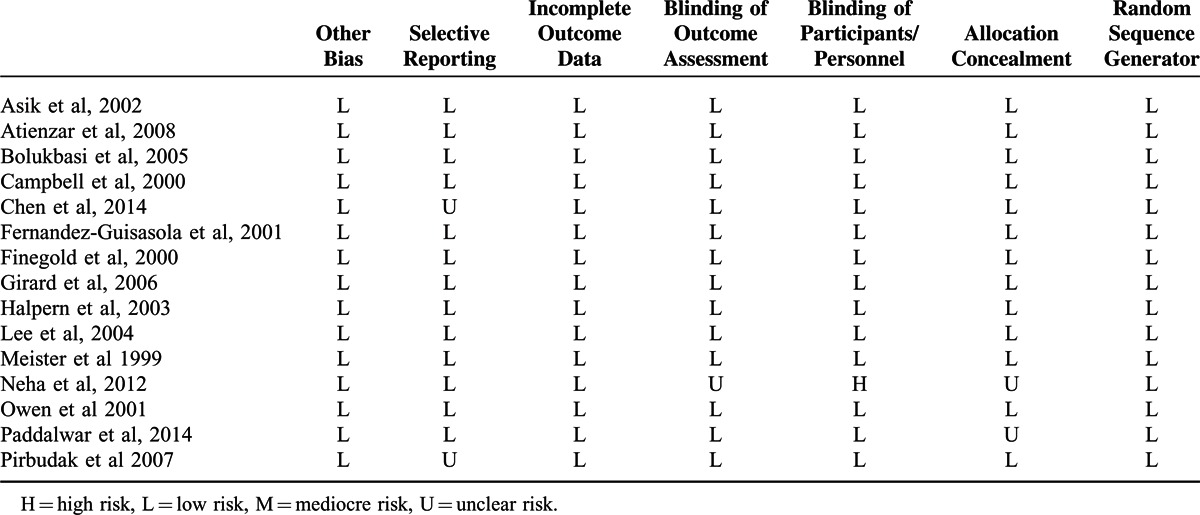
Risk of Bias Assessment in the Included Studies

There were no statistically significant differences between both the combinations in the mean change in VAS for pain during labor, incidence of instrumental or cesarean deliveries, duration of either first or second stage of labor, neonate Apgar score < 7, maternal satisfaction with analgesia, oxytocin use of induction, onset of analgesia, and duration of analgesia (Table 4).

**TABLE 4:**
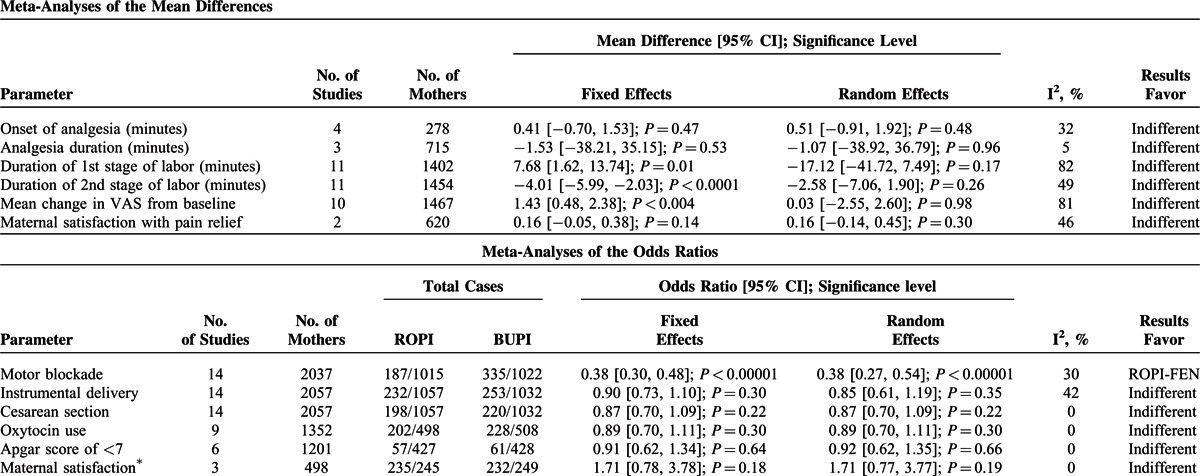
Major Findings of the Meta-Analyses of Various Parameters

**FIGURE 3:**
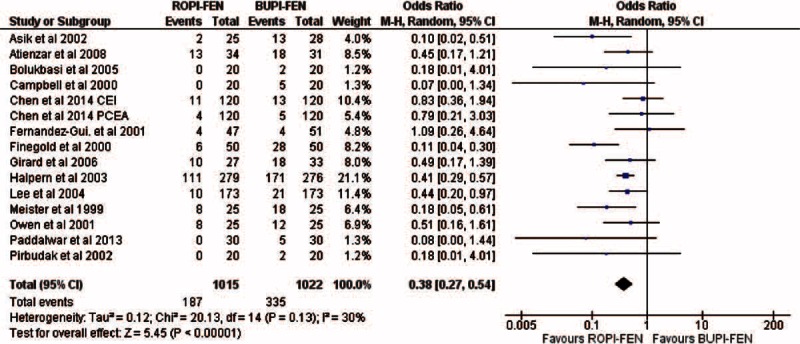
Forest plot showing significantly lower incidence of motor blockade in epidural ROPI-FEN administered women in the meta-analysis under random effects model. In Chen et al, 2014, PCEA = patient control epidural analgesia, CEI = continuous epidural infusion.

In the overall study population, 187 of 1015 women in ROPI-FEN group and 335 of 1022 women in BUPI-FEN group developed notable motor blocks as measured by modified Bromage scores given in Table 1. Both the odds ratio-based models revealed ROPI-FEN group to be significantly superior to BUPI-FEN combination (OR [95% CI] of 0.38 [0.27, 0.54], *P* < 0.00001, REM; and 0.38 [0.30, 0.48], *P* < 0.00001, FEM; I^2^ 30%, Figure 3).

Incidence of motor blocks increased significantly with increasing concentration of bupivacaine (correlation coefficient [*r*] = 0.566, *P* = 0.027 but not significantly with ropivacaine 0.378, *P* = 0.164). Overall, there were no significant relationships between fentanyl concentration and the incidence of motor blocks (*r* = 0.153, *P* = 0.58 for BUPI-FEN and *r* = 0.215, *P* = 0.44 for ROPI-FEN). However, with increasing concentration of fentanyl from 0.0001% to 0.00025% (all studies except one which used 0.0005% fentanyl),^23^ the percent incidence of motor blockade decreased significantly in ROPI-FEN (*r* =  − 0.549; *P* = 0.034) but not significantly in BUPI-FEN (−0.284, *P* = 0.3) treated women.

Increasing dose concentrations of either local amide anesthetics or fentanyl were not associated with an increased incidence of instrumental or cesarean deliveries. However, with increasing concentration of fentanyl from 0.0001% to 0.00025% (all studies except one with 0.0005%),^23^ the percent incidence of instrumental deliveries decreased significantly in ROPI-FEN (*r* = −0.532, *P* = 0.04) but not significantly in BUPI-FEN (*r* = −0.185, *P* = 0.51) treated women.

Subgroup analyses were performed to assess the interstudy dose concentration deviation. In the meta-analyses of the studies in which bupivacaine and ropivacaine concentrations of either ≥0.125% or <0.125% were used, the results did not differ significantly from the overall results for all parameters studied.

Statistical heterogeneity was high in 2 comparisons: I^2^ was 81% in the meta-analysis of the mean change in VAS for pain, however, sensitivity analysis (exclusion of 1 study^23^) reduced I^2^ to 38% level without any significant difference on overall outcomes. In the comparison of the duration of first stage of labor, I^2^ was 82%. Sensitivity analysis (exclusion of 1 study^24^) reduced I^2^ value to 7% level with outcomes still nonsignificantly different between the comparator combinations.

Side effects associated with the combinational use of these amide local anesthetics in combination with fentanyl noticed in the included studies were pruritus, nausea, and hypotension which were observed in at least 4 studies. Percent incidence was similar in BUPI-FEN versus ROPI-FEN groups (pruritus 29.9% ± 24.5% versus 31.25% ± 20.68%; nausea 7.57% ± 5.6% versus 7.39% ± 6.48%; and hypotension 11.7% ± 11% versus 13.12% ± 16.49%). Besides, backache (10%), shivering (5%), and fetal bradycardia (10%) were also observed in 1 study each. Correlation coefficient between the percent incidence of pruritus and fentanyl concentration was 0.36, *P* = 0.27 for BUPI-FEN and 0.34, *P* = 0.3 for ROPI-FEN combinations.

## DISCUSSION

Several measures of efficacy and safety were examined in the present meta-analysis, and majority of these were found comparable. There were no significant differences in the mean change in VAS for pain during labor, incidence of either instrumental or cesarean deliveries, duration of either first or second stage of labor, neonate Apgar scores of <7, maternal satisfaction with analgesia, oxytocin use for induction, duration of analgesia, and onset of analgesia between both the combinations. However, the incidence of motor blocks was significantly lower in ROPI-FEN-administered women. Percent women who developed motor block measurable with Bromage scale were 18.4% in ROPI-FEN and 32.8% in BUPI-FEN treated groups.

It is postulated that ropivacaine possesses low lipophilic characteristics and therefore is resistant to rapidly penetrating the myelinated nerve fibers and thus is less likely to cause motor blockade and neurotoxicity.^30^ However, in a meta-analysis, Lv et al^10^ could not find a significant difference in the incidence of motor blocks between bupivacaine−sufentanil and ropivacaine−sufentanil combinations but noted a significantly higher incidence of instrumental deliveries in ropivacaine−sufentanil treated women (*P* = 0.05), although, the percent incidence of motor blocks was slightly higher in bupivacaine−sufentanil treated women.

Timing of the incidence of motor blocks can also affect the overall outcomes of the labor analgesia as seen in one of the included studies of the present meta-analysis in which all motor block events initiated in the first 3 hours of labor in ROPI-FEN and within 4 hours of BUPI-FEN treated women. This was in accordance with the incidence of instrumental deliveries (9 ROPI-FEN vs 14 BUPI-FEN).^21^ A similar difference between these comparators in the timeline of motor block incidence was observed by Halpern et al^23^ but this was in accordance with the incidence of cesarean rather than instrumental deliveries.

In order to seek a causal relationship between the incidence of motor blocks and ropivacaine use, it was speculated that lesser ropivacaine use on hourly basis may be a cause of low incidence of motor blocks,^25^ but such an association could not be observed in the present study. However, in the present study, all except 4 studies (0.125% vs 0.2%,^16^ 0.08% vs 0.1%,^19^ 0.0625% vs 0.1%,^20^ and 0.125% vs 0.1%^21^) used same concentrations of bupivacaine and ropivacaine, but still the incidence of motor blocks was higher in bupivacaine group. Moreover, correlational association of motor blocks was significant with bupivacaine but not with ropivacaine. These results favor the notion that motor blockade is a drug effect and cannot be attributed to potency.^31^ It has been previously demonstrated that ropivacaine possesses up to 40% lower potency relative to racemate bupivacaine.^32^

Incidence of motor block can prolong the second stage of labor leading to more chances of instrumental delivery.^33^ Analgesia with the combination of low dose opioid and local anesthetic has been suggested to cause lower incidence of instrumental deliveries.^34–36^ The present study favors this notion conditionally as increasing concentration of fentanyl from 0.0001% to 0.00025% was associated with decreased incidence of motor blocks and instrumental deliveries, but a statistically significant effect was observed only for ropivacaine and fentanyl combination. This significant relationship no longer existed when a study that utilized 0.0005% fentanyl was included in the correlational analysis. This may indicate that fentanyl concentrations below 0.0003% may serve as optimal dose concentrations in combination with about 0.1% ropivacaine for labor. However, more data will be required to test such a hypothesis.

In the present study, the incidence of side effects, other than motor blocks, was similar between the comparator groups. Notably, the incidence of pruritus was about 30% in each combination. In the meta-analysis of Lv et al,^10^ the percent incidence of pruritus was also similar (BUPI-sufentanil 31% vs ROPI-sufentanil 35%). Similarly, the incidence of pruritus was observed as 36% in BUPI-FEN and 40% in BUPI-sufentanil groups in a meta-analytical review which attempted to study the efficacy and safety of these combinations.^37^ Thus, in these 3 reviews, the incidence of pruritus was 30% to 36% in fentanyl groups and 31% to 40% in sufentanil groups when used in combination with local amides.

This meta-analysis synthesizes data from 15 studies (over 2000 participants) and for most of the comparisons, statistical heterogeneity was low or moderate. Among the limitations of this analytical review, a factor with mild effect can be that some of the included studies used loading doses of anesthetics other than bupivacaine–fentanyl or ropivacaine–fentanyl, which might have slight impact on motor function; however, such an impact would have been shared by both the groups. Variations in loading volumes and timing, and PCEA locktime and volume may also have slight impact on the overall outcomes. There can be some impact of unaccountable confounding factors in the correlative analyses of the present study. The available data did not allow performing meta-regression to further explore such relationships.

## CONCLUSION

Ropivacaine in combination with fentanyl at 0.1:0.0002 ratio for labor epidural analgesia is associated with significantly lower incidence of motor blocks besides exhibiting comparable analgesic properties to that of bupivacaine with fentanyl (0.1:0.0002) as seen in several parameters including onset of analgesia, mean change in VAS for pain during labor, Apgar scores of less than 7, oxytocin use for induction, first and second stage of labor, incidence of instrumental or cesarean delivery, and maternal satisfaction. Safer toxicity profile of ropivacaine in combination with fentanyl favors its use, especially in conditions where motor blockade can be a stronger risk factor.
